# Upper Limb Electromyographic Responses to Motor Imagery and Action Observation in Acquired Brain Injury

**DOI:** 10.3390/s24061802

**Published:** 2024-03-11

**Authors:** Sara Santiago-Martín, Ana Belén Calvo-Vera, Beatriz María Bermejo-Gil, Ana María Martín-Nogueras

**Affiliations:** 1 Salamanca Association of Acquired Brain Injury (ASDACE), 37007 Salamanca, Spain; 2 Department of Nursing and Physiotherapy, University of Salamanca, 37007 Salamanca, Spain; abcvera@usal.es (A.B.C.-V.); beatriz.bermejo@usal.es (B.M.B.-G.); anamar@usal.es (A.M.M.-N.); 3 NeuroUsal Team, 37007 Salamanca, Spain; 4 Salamanca Biomedical Research Institute (IBSAL), 37007 Salamanca, Spain; 5 Rehabilitation Service, University Hospital of Salamanca, 37007 Salamanca, Spain; 6 Institute of Neurosciences of Castilla y León (INCYL), 37007 Salamanca, Spain

**Keywords:** stroke, surface electromyography (sEMG), upper extremity, occupational therapy

## Abstract

Acquired Brain Injuries are one of the leading causes of mortality and disability worldwide. One of the most frequent sequelae is motor impairment of the upper limbs, which affects people’s functionality and quality of life. Following the discovery of mirror neurons, new techniques were developed based on the mechanisms of neuronal plasticity, such as motor imagery (MI) and action observation (AO). We propose a protocol using electromyographic recordings of forearm muscles in people who have suffered a stroke during an MI task and an AO task. Three different experimental conditions will be studied during the electromyographic recordings: control recording, recording during MI, and recording during AO. Understanding the muscle activation in each technique will allow us to develop future protocols and intervention plans, improving the quality of care for people who have suffered a stroke.

## 1. Introduction

Acquired Brain Injury (ABI) is defined as a sudden injury to the brain, classified as an ischemic stroke (blockage of a blood vessel) or haemorrhagic stroke (rupture of a blood vessel) [1]. The increases in life expectancy and healthcare quality have increased the number of survivors affected by stroke [2,3]. This situation will increase costs associated with service provision and loss of productivity, making studying rehabilitative techniques for improved functional recovery essential.

People who have suffered a stroke may have physical, cognitive, emotional, and social sequelae that alter their functionality and quality of life. Many of these people suffer motor impairments at the level of the upper limbs, which implies severe difficulties in performing activities of daily living (ADLs) [4]. Improving the indication of treatment tools will allow better results to be achieved in increasing these people’s functionality and quality of life.

Rizzolatti’s discovery of mirror neurons in the 1990s was a significant breakthrough in neurorehabilitation [5], as new tools and therapeutic approaches based on neuroplasticity mechanisms appeared. In these tools, the focus is on achieving more direct brain stimulation, achieving motor relearning through the mechanisms of neuronal plasticity. Motor imagery (MI) and action observation (AO) are some of the techniques in this approach. In contrast, other more traditional techniques derived from the bottom-up approach seek to act at the physical level and produce changes at the central nervous system level, such as Vojta or Bobath therapy [6].

MI involves the cognitive task of imagining the realisation of the movement without physically executing it. It is based on the use of cognitive and perceptual processes that results in neural activation similar to that which occurs when the actual execution of the movement or task is performed [7]. Its efficacy in activating cortical and subcortical areas has been demonstrated [8]. However, more evidence must be presented regarding the motor response it elicits. In addition to knowing that the brain areas that are activated during MI are areas related to motor planning, programming, and execution [9], it is of great interest to know the degree of muscle activation and what individual characteristics or determinants modify it during neurorehabilitation.

On the other hand, AO is another rehabilitation tool that facilitates the activation of the motor cortex by activating the mirror neuron system [10]. Studies show that AO facilitates the excitability of the motor system, i.e., facilitates movement execution and motor learning [11]. This tool consists of watching a video showing movements or activities performed in the first or third person. Subsequently, the patient is usually asked to perform the action that has been observed, reinforcing the activation that has occurred at the cortical level.

Studies have focused on the encephalographic activity produced when these techniques are applied [12]. For example, in the case of MI, several studies claim that the frontoparietal, subcortical, and cerebellar regions; the anterior intraparietal cortex; the primary motor cortex; the bilateral supplementary area; and the premotor area are activated [13]. However, the scientific evidence for muscle activation in AO and MI is limited. Other studies confirm that techniques such as MI in neurorehabilitation could be very beneficial because mentally evoking specific movements could lead to functional reorganisation [14].

Most studies focus on the acute phase of the disease. However, analysing what muscle activation occurs in the chronic phase is of great interest. Furthermore, AO and MI are low-cost, easy-to-apply alternatives when other techniques are not possible. When implementing these techniques, it is essential to know the mental evocation capacity of the patients, i.e., their ability to generate mental images. This knowledge is essential as the benefits of these techniques are directly related to this ability [15]. Tools such as the Mental Evocation of Images, Movements and Activities Questionnaire (CEMIMA) can be used to assess mental imagery capacity in individuals with an ABI and evaluate the effectiveness of the applied techniques.

Assessing the muscular response when AO and MI are performed, as well as the individual characteristics of the subjects, will allow us to propose future protocols for stroke patients with motor impairments.

The primary objective of this study is to record muscle activation in the affected upper limb during motor imagery and action observation in people who have suffered a stroke.

The secondary objectives are as follows:

- To describe the muscle activation in the wrist and finger flexors and extensors that occurs during the use of the CEMIMA as a motor imagery tool via surface electromyographic recordings.
- To describe, using surface electromyographic recordings, the muscle activation in the wrist and finger flexors and extensors that occurs during the application of action observation.
- To analyse whether there are differences between the subjects who evoke better or worse mentally depending on their electromyographic response in MI and AO.
- To analyse the association between the degree of independence in ADLs, upper limb functionality, quality of life, mental evocation, and cognitive impairment with the electromyographic response during MI and AO.
- To analyse, from a gender perspective, the determinants of the study in men and women.
- To study whether there is a relationship between the electromyographic response during MI and AO by age or age groups (over 65 years).

## 2. Experimental Design

### 2.1. Type of Study

A quasi-experimental study will be conducted on a convenience sample under three different experimental conditions.

### 2.2. Study Setting

The study will be conducted at the Faculty of Nursing and Physiotherapy of the University of Salamanca starting 1 January 2024. The protocol for this study was approved by the Salamanca Health Area Drug Research Ethics Committee on 25 September 2023 (CEIm Ref. 2023/09) and follow the guidelines of the Helsinki Declaration. The study is registered at ClinicalTrials.gov, ID: NCT06230718

### 2.3. Participants

The study will be carried out on a population of adults with stroke. Subjects will be recruited by convenience sampling through the Rehabilitation Service of the University Hospital of Salamanca and the Salamanca Association of Acquired Brain Injury (ASDACE). Subjects will be asked to participate voluntarily in the study, and once the objectives and content have been explained, they will sign the corresponding informed consent form.

The inclusion criteria are:

- Being over 18 years of age with an established diagnosis of stroke.
- Presenting motor response limitations or deficiencies in the upper limbs.
- Maintaining cognitive functions with the capacity to follow the indications of the interventions and assessments in Spanish language (The Montreal Cognitive Assessment, MoCA > 14).

The following exclusion criteria will be considered: having been diagnosed with mental illness before the stroke; presenting with central nervous system conditions other than stroke or severe or acute cardiorespiratory system disorders; and presenting with fever, muscle inflammation, or myopathies, as well as any vascular and organic insufficiency, liver disease, or skin lesions in the area of application of the electrodes.

### 2.4. Power Calculation and Sample Size

It is estimated that to conduct a study using linear regression tests for each predictor, considering an effect size between 0.5 and 0.8, a statistical power of 90%, and a safety level of 95% and taking into account the variance of a previous pilot study conducted, between 21 and 28 subjects will be required.

## 3. Materials and Equipment

### 3.1. Variables Study

All study variables are shown in Table 1. In the first column, the baseline variables, including socio-clinical-demographic data of all participants, are shown. The second column presents the primary variables of the study.

### 3.2. Measurement Instruments

1. Degree of disability and dependence: Barthel Index. This is a generic measure that assesses the level of independence of the patient, allowing the degree of performance of the subject in some basic activities of daily living to be determined, assigning different scores and weightings. It is the most widely used scale internationally for the functional assessment of patients with an acute cerebrovascular pathology and its complications, such as vascular dementia. It includes 10 ADLs: eating, transferring between chair and bed, personal grooming, going to the toilet, bathing/showering, transferring (walking on a smooth surface or in a wheelchair), climbing/descending stairs, dressing, stool control, and urine control [16].
2. Functionality of the upper limbs: Action Research Arm Test (ARAT). This test determines the functionality of the upper limbs after a cortical injury by assessing the ability to manipulate objects of different sizes, weights, and shapes with both hands. It consists of 19 tests divided into four subscales: gross grasp, grasping, pinching, and gross movement. The ARAT takes between 5 and 15 min to administer. It is a reliable and valid measure to assess the motor function of a person who has suffered a stroke. It also allows an assessment of spontaneous recovery and can detect changes that, after intervention, are in the range of clinically significant values [17].
3. Cognitive impairment: Montreal Cognitive Assessment (MoCA). A valid instrument for assessing mild cognitive dysfunction. It consists of the following domains: attention, concentration, executive functions (including the capacity for abstraction), memory, language, visoconstructive abilities, calculation, and orientation. The maximum score is 30. A score of ≥26 is considered normal. Administration time: 10 min [18].
4. Loewenstein Occupational Therapy Cognitive Assessment (LOTCA). This is a standardised occupational-therapy-specific test for use with stroke patients. The LOTCA consists of 20 subtests and assesses four areas: orientation, visual and spatial perception, visual motor organisation, and thinking operations. This test suite is consistent, internally valid, and reliable for determining an initial profile of the cognitive and perceptual abilities of the brain-injured patient [19].
5. Mental evocation capacity: Mental Evocation of Images, Movements and Activities Questionnaire (CEMIMA). The questionnaire consists of two subscales: one of evocation and the other of sensation. It measures the ability to mentally form visual and kinaesthetic images of the hand/upper limb and can also be used to assess the imagination process of the person being assessed. A study of its reliability and validity determined it as an instrument that allows measurements of the ability to mentally form visual and kinaesthetic images of the hand/upper limb and allows an understanding of the imagination process of the person evaluated [20].
6. Perception of quality of life: Stroke Impact Scala 16 (SIS-16). This is a self-report comprising 16 items from four physical domains (strength, hand function, mobility, and ADL/instrumental activities) included in the SIS 3.0. This facilitates its application, as it can be applied in a shorter time, in around 5–10 min [21].

### 3.3. Electromyographic Equipment

This study will require a Surface Electromyographer, a bioamplifier (EMG Brainquiry Personal Efficiency Trainer^®^: PET 4, Brainquiry, Nijmegen, The Netherlands) and pre-gelled adhesive electrodes with a hook-and-loop of 18 mm. Furthermore, it requires a chair for the patient, a table, and a screen to display videos. The recordings will be digitised using 55 Hz high-pass filtering and stored in raw form. The Average Rectified Value (ARV) and Root Mean Square (RMS) of the obtained data will be determined for amplitude, and the Median Frequency (MNF) and Mean Power Frequency (MDF) will be determined for frequency. Subsequently, the electromyographic power of the target contractile events will be assessed after discrimination of possible recording interferences and artefacts. The contractile activity shall be considered when the electromyographic signal exceeds the mean plus two standard deviations in the baseline activity of each muscle for at least 100 ms.

### 3.4. Videos

**Figure 1:**
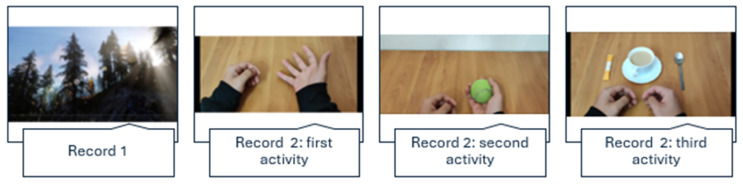
Records 1 and 2.

Two recordings of identical durations will be available to compare the EMG recordings under each condition. Recording 1 will show images of landscapes without the presence of any person or animal and will be used for the control condition. Recording 2 will be based on the activities of the CEMIMA. In this recording, three activities will be visualised: opening and closing the hand, throwing and catching a tennis ball, pouring sugar into a coffee cup and stirring (Figure 1). The first minute of both recordings will serve as the resting time, which is essential for later analysis of the results. Recordings will be available in the open access repository for the research community once the study is finished.

## 4. Detailed Procedure

### 4.1. Experimental Conditions

**Figure 2:**
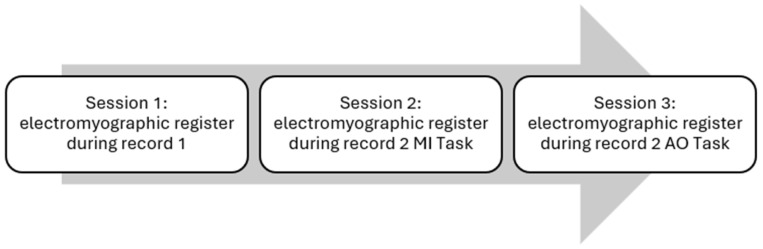
Schedule of experiments.

The study participants will take part in three sessions, one session per week, and in each one, different experimental conditions will be used: experimental control conditions, experimental MI conditions and experimental AO conditions (Figure 2). In the first session, the socio-clinical-demographic data of all participants will be collected.

Recording 1 will be used during the control experiments and Recording 2 will be used in the MI and AO experiments (Figure 2); in the first case, the Spanish audio of the recording will be used, and in the second case, the images will be used. The first minute of both recordings will serve as a resting time, which is essential for subsequent analysis of the results.

During all three experiments, subjects will be seated in front of a stretcher on which they will rest their forearms with their elbows in slight flexion. Before the first experimental condition, socio-clinical-demographic data will be collected for the different assessment tools.

### 4.2. Electromyographic Recording

**Figure 3:**
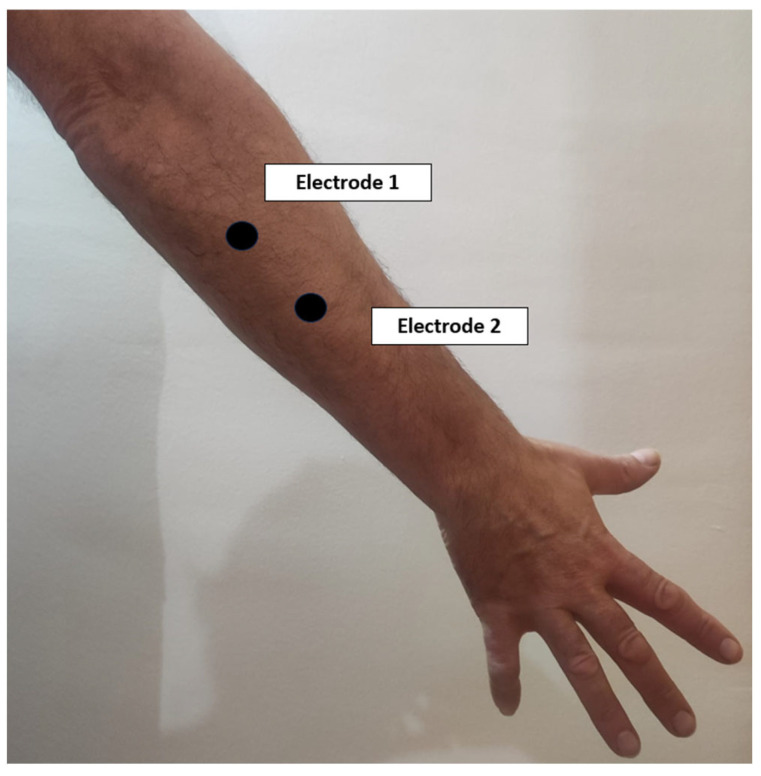
Electrode placement scheme for extensor muscles.

**Figure 4:**
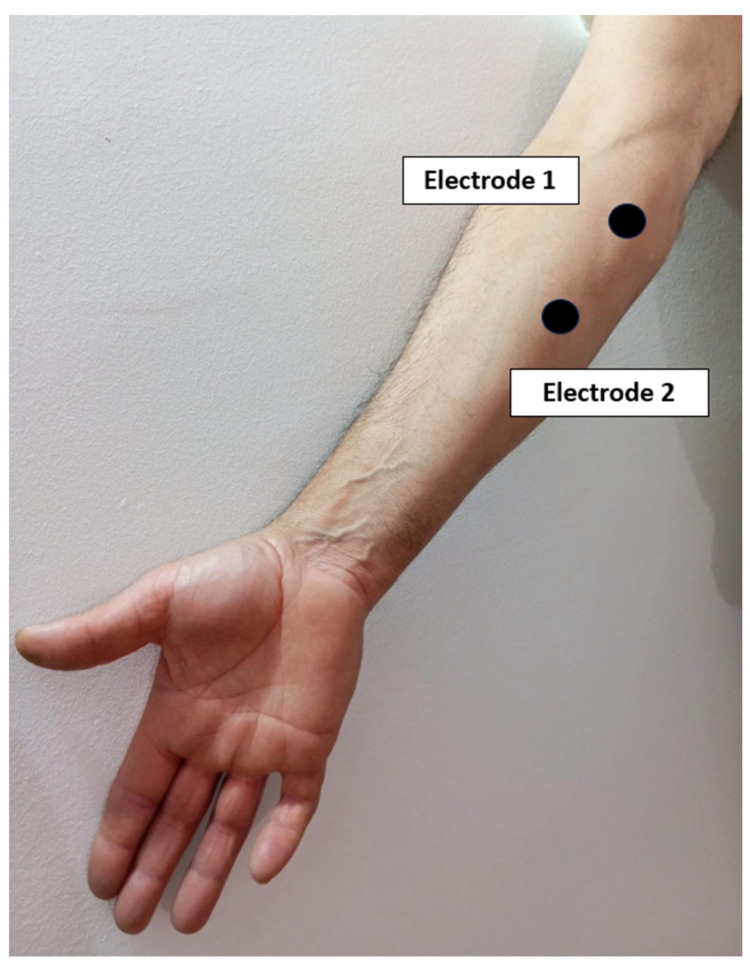
Electrode placement scheme for flexor muscles.

Pre-gelled adhesive bipolar electrodes will be placed by feeling the muscle and considering the direction of the muscle fibres according to Abarrotar’s criteria [22]. The same electrode positions will be used during all recording sessions:

- The ground electrode will be placed on the clavicle.
- The electrodes corresponding to the first channel will be placed on the common extensor digitorum on the posterior aspect of the forearm, with a distance between them of approximately 2 cm (Figure 3).
- The electrodes corresponding to the second channel will be placed on the common deep flexor digitorum on the anterior aspect of the forearm, with a distance between them of approximately 2 cm (Figure 4).
- The reference electrode will be placed over the biceps brachii.

### 4.3. Data Analysis

The data will be entered into a database, where each subject will be assigned a number and will not be linked to any data that could identify them, guaranteeing their anonymity. Data analysis will be performed by an external statistician using SPSS Statistics for Windows v.28.0 (IBM Corp, Armonk, NY, USA). The most appropriate tests will be established according to the characteristics of the distributions such as descriptive, correlational, and multivariate analyses. An alpha value of 0.05 is established as the level of significance.

## 5. Expected Results

This study will test whether MI and AO activate flexor and extensor muscles of the wrist and fingers in stroke patients. Furthermore, it will try to determine if this activation is related to different factors such as image evocation, the degree of independence in daily life activities, the functionality of upper limbs, age, or gender.

### 5.1. Strengths and Weaknesses of the Study

The strong point of this study is the systematisation of an electromyographic recording protocol of the upper limbs, which allows us to determine whether specific neurorehabilitation tools provoke activation not only at the cortical level but also as a natural response at the muscular level. This is fundamental, as the literature shows the need for more research on EMG analysis in the upper limbs, as well as the need to establish a methodology and a standardised protocol [23].

Previous studies have shown the activation of different brain areas during MI: the precentral gyrus of the primary motor cortex [24], the premotor area [25], the corpus callosum [26], the parietal cortex [27], the cerebellum [28], and the basal ganglia [29]. During AO, Broca’s area and the premotor cortex [30], the inferior parietal cortex [31], the superior temporal cortex [32], the superior parietal cortex [33], the temporoparietal junction [34], and the basal ganglia [35] are activated. Knowing what the muscular response is in people who have suffered a stroke will allow us to improve the therapeutic indications for these patients.

Furthermore, knowing whether the muscular activation caused by MI or AO is related to the mental evocation capacity, the degree of independence in ADLs, upper limb functionality, quality of life, cognitive impairment, gender, or age will allow us to adopt the most appropriate intervention in each case and thus achieve the best functional recovery.

On the other hand, scientific evidence of the benefits of neurorehabilitation treatment tools focused on the person with stroke will increase the empowerment and safety of patients, as well as their perspective and that of their family towards treatments.

Another strength of this protocol is that it has taken into account certain aspects that have already been demonstrated in other studies. Repeatedly imagining different movements can lead to mental fatigue, affecting motor performance [36]. Therefore, it is essential to control the duration of the recordings and the number of movements and repetitions when designing protocols. In the case of AO, protocols and studies that have been carried out using EMG have shown that muscle activation occurs, and, after training, changes in the contraction of the muscles were studied [37,38]. However, these studies have not been conducted in the therapeutic field, so demonstrating that protocols of this type are effective may open doors in neurorehabilitation.

In terms of limitations, the main limitation may be the number of muscles evaluated, limited by the recording system to be used. It would be interesting to be able to record electromyographic activity in the contralateral limbs. Another limitation may be the convenience sample and its regional context. Furthermore, the heterogenicity of strokes could impact data generalisation. As it is an exploratory intervention, participants adherence can be influenced.

Finally, the analysis of the correlation between the electromyographic response by age may be limited by the smaller number of subjects expected due to the prevalence of stroke in those under 60 years of age.

### 5.2. Contribution to Occupational Therapy and Physiotherapy

Scientific knowledge in the disciplines of health sciences has been linked to academic recognition in different parts of the world. Until ten years ago, in Spain, physiotherapists, occupational therapists, and nurses were not able to start a research career by a purely natural route. This has happened in other parts of the world, and in some countries today, physiotherapists and occupational therapists are not recognised as having the ability to perform research. The results of this study will contribute to the development of these disciplines in the field of neurorehabilitation.

### 5.3. Ethics and Dissemination

The study is authorised by the Ethics Committee for Research with Medicines of the Salamanca Health Area and follows the principles of the Declaration of Helsinki. All subjects will be informed of the objectives of the project and the risks and benefits of the examinations to be performed and will sign a corresponding informed consent form. No risk or discomfort is foreseen with applying any of the experimental conditions.

All the information on this study will be coded and anonymised according to the corresponding Data Management Plan. The informed consent forms will be kept in the facilities of the NEURORREHABILITACION (APSF-19)-NEUROUSAL research team following current legislation (Organic Law 3/2018 of 5 December on Personal Data Protection and Guarantee of Digital Rights and Regulation (EU) 2016/679 of the European Parliament and of the Council of 27 April 2016 on Data Protection, RGPD).

## Figures and Tables

**Table 1:** Study variables.

Baseline Variables	Primary Variables
Date of birth Gender Marital Status Level of Education Economic Level Affected Side Dominant Side Type of Injury Time Since Injury Functionally of upper limbs Cognitive impairment	Degree of disability and dependence Cognitive and perceptual impairment Mental evocation capacity Perception of quality of life Surface electromyography

## Data Availability

The raw data supporting the conclusions of this article can be made available by the authors upon valid request to the corresponding author.
